# Ion-Exchange Membranes and Processes (Volume II)

**DOI:** 10.3390/membranes11110816

**Published:** 2021-10-26

**Authors:** Natalia Pismenskaya, Semyon Mareev

**Affiliations:** Membrane Institute, Kuban State University, 350040 Krasnodar, Russia; mareev-semyon@bk.ru

Ion exchange membranes (IEMs) and related processes have generated increased interest among researchers in the last few years, according to the analysis of publication activity in Scopus. This is primarily due to the emergence of new ion-exchange materials, as well as strategies and technologies for the production of ion-exchange membranes. The application of low-priced fillers that simultaneously serve as an inert binder and a reinforcing material, the use of inorganic and mixed organic and inorganic materials, and the surface modification of commercial IEMs have become drivers for improvements in transport characteristics, reducing fouling and lengthening the lifecycle of membranes, as well as providing high permselectivity to singly charged counterions.

Moreover, researchers have paid particular attention to anion-exchange membranes, which have vital characteristics for hydrogen generation systems and relatively new applications for blue energy production such as as reverse electrodialysis (RED), the ability to process liquid media in the food and pharmaceutical industries, and the ability to extract valuable components, for example, lithium, from various waste and natural waters using dialysis and electrodialysis. The Special Issue “Ion-Exchange Membranes and Processes II” highlights these trends.

McHugh et al. [1] synthesized an anion-exchange membrane consisting of a fluorinated polymer backbone grafted with imidazole and trimethylammonium units, and investigated its chemical composition, structure, exchange capacity, electrical conductivity, mechanical strength, and electrochemical characteristics. They compared the properties of the new membrane with those obtained for commercial Fumapem and PVIB-10 membranes and concluded that, in the long term, the ion-exchange material could be deve-loped into a cost-effective conductive separator for electrolyzers, providing simultaneous hydrogen generation and lignin oxidation.

Khan et al. [2] reported on the synthesis of a dimethylethanolamine-grafted anion-exchange membrane by incorporating dimethylethanolamine as the ion-exchange content into the polymer matrix via the solution-casting method. This membrane exhibited a homogeneous morphology, water uptake of 115%, an ion-exchange capacity of 2.70 meq/g, and high thermal stability. The authors obtained sorption isotherms and concluded that the new membrane could be employed as an extraordinary candidate for the removal of methyl orange from an aqueous solution.

Another article by Khan et al. [3] focused on the synthesis of a series of functionalized inorganic/organic composite anion-exchange membranes, containing varying amount of inorganic filler consist of N-(trimethoxysilylpropyl)-N,N,N-trimethylammonium chloride in a quaternized poly (2, 6-dimethyl-1, 4-phenylene oxide) matrix. The optimal ratio of organic and inorganic phases in the composite was found by analyzing the ion-exchange capacity, the water uptake, and the chemical and mechanical stability of the prepared samples. The best samples demonstrated a high separation factor of 139–260 during diffusion dialysis of a solution imitating wastewater containing Cl^−^ and FeCl_2_.

Cation-exchange membrane properties were analyzed by Sarapulova et al. [4] and Falina et.al. [5]. The study in [4] provides a comparative analysis of the characteristics of the new cation-exchange membranes CJMC-3 and CJMC-5 (Hefei Chemjoy Polymer Material Co. Ltd., China), along with the characteristics of the well-known commercial CMX membrane (Astom, Japan). The conductivity in NaCl, Na_2_SO_4_, and CaCl_2_ solutions, the diffusion permeability, the counterion transport numbers, and the current–voltage cha-racteristics in NaCl solutions were also considered, as well as water splitting and fouling by polyphenols. Sarapulova et al. recommended CJMC-3 and CJMC-5 for use in the processing of natural and industrial wastewater via electrodialysis for the removal of large ions or the treatment of food liquids: juices, wine, milk products, etc.

The authors in [5] discuss the applicability of homogeneous and heterogeneous ca-tion-exchange membranes for the separation via electrodialysis of the monovalent/divalent cations Na^+^/Ca^2+^ or H^+^/Ca^2+^. These membranes were directly modified by polyaniline in the electrodialysis cell. Both modified membranes showed a significant decrease in diffusion permeability and high permselectivity for singly charged cations. It was shown for the first time that polyaniline-modified cation-exchange membranes have permselectivity to protons in both underlimiting and overlimiting current modes.

Perreault et al. [6] evaluated the suitability of homogeneous and heterogeneous cation-exchange membranes with an aromatic or aliphatic ion-exchange matrix, for use in the processing of cranberry juice via electrodialysis. Their experiments showed that MK-40 (Shchekinoazot, Russia), CEM Type-II (Fujifilm, The Netherlands), and CSE-fg (Astom, Japan) were more prone to fouling than CJMC-5 (Hefei Chemjoy Polymer Material Co. Ltd., China) due to their high ion-exchange capacity, thickness, and the presence of meso and macropores in the structure. At pH 10, the desorption solution allowed for a better recovery of anthocyanins, while at pH 6 a better extraction of proanthocyanidins was achieved from the cation-exchange membranes under study.

Mehdizadeh et al. [7] tested the effectiveness of the new asymmetric monovalent selective membranes CIMS and ACS-8T (Astom, Japan) in the RED process for harvesting energy from the salinity gradient between two solutions. Natural and model reverse osmosis brines, as well as seawater, were used as the high-concentrate feed solutions, and river water was used as the low-concentrate feed solution. The authors concluded that the application of membranes that are selective to singly charged counterions increases the power output due to the decrease in the negative impact on the process of multivalent counterions contained in the natural feed solutions. 

Gao et al. [8] developed a bipolar membrane electrodialysis technology to treat waste sodium sulfate containing lithium carbonate. This environmentally friendly technology allowed for the conversion of the low-value sodium sulfate into high-value sulfuric acid and sodium hydroxide, with a conversion rate close to 100%. The purity of these products was 98.3% and 98.2%, respectively.

## Data Availability

Not applicable.
